# Short-term environmental enrichment modulates brain region-specific redox responses in adolescent female mice

**DOI:** 10.3389/fnagi.2026.1889960

**Published:** 2026-08-31

**Authors:** Matheus Santos de Sousa Fernandes, Alexandre Kanashiro, Tiago Lacerda Ramos, Danielle Dutra Pereira, Vanessa Lima de Souza, Claudia Jaques Lagranha, Sacide Tüfekci, Burak Yagin, Nouf H. Alkhamees, Fabrício Oliveira Souto

**Affiliations:** 1 Keizo Asami Institute, Federal University of Pernambuco, Recife, Brazil; 2 Graduate Program in Applied Biology to Health, Federal University of Pernambuco, Recife, Brazil; 3 Department of Pharmacology, Ribeirão Preto Medical School, University of São Paulo, Ribeirão Preto, Brazil; 4 Center for Research in Inflammatory Diseases, Ribeirão Preto Medical School, University of São Paulo, Ribeirão Preto, Brazil; 5 Department of Histology and Embryology, Federal University of Pernambuco, Recife, Brazil; 6 Laboratory of Biochemistry and Molecular Biology of Physical Exercise, Academic Center of Vitoria De Santo Antão, Federal University of Pernambuco, Vitória De Santo Antão, Brazil; 7 Department of Sport Management, Faculty of Sport Sciences, Kahramanmaras Sutcu Imam University, Kahramanmaras, Türkiye; 8 Department of Biostatistics and Medical Informatics, Faculty of Medicine, Inonu University, Malatya, Türkiye; 9 Department of Rehabilitation Sciences, College of Health and Rehabilitation Sciences, Princess Nourah Bint Abdulrahman University, Riyadh, Saudi Arabia

**Keywords:** antioxidant defense, brain regional heterogeneity, enriched environment, female mice, oxidative balance

## Abstract

**Background:**

Environmental enrichment (EE) is a non-pharmacological intervention known to enhance neuroplasticity and resilience. However, studies of EE on brain redox balance have focused predominantly on males and single regions, leaving the developing female brain and region-specific responses largely uncharacterized. This study investigated the effects of a three-week EE protocol on oxidative balance in the hippocampus, hypothalamus, and brainstem of adolescent female C57BL/6 mice.

**Methods:**

Lipid peroxidation was measured by thiobarbituric acid reactive substances (TBARS), and protein oxidation by carbonyl content. The enzymatic antioxidant system was evaluated through the activities of superoxide dismutase (SOD), catalase (CAT), and glutathione S-transferase (GST). Non-enzymatic antioxidant defenses included quantification of reduced glutathione (GSH), oxidized glutathione (GSSG), the GSH/GSSG ratio, and total sulfhydryl content, providing an integrated assessment of redox homeostasis.

**Results:**

In the hippocampus, EE promoted a dual redox profile characterized by increased TBARS alongside reduced protein carbonylation, increased SOD activity, decreased GSSG, and elevated total sulfhydryl content. In the hypothalamus, EE exerted a protective effect, reducing both lipid and protein oxidation without altering enzymatic antioxidant activities. Instead, non-enzymatic defenses were selectively enhanced, as evidenced by increased GSH and sulfhydryl levels. In the brainstem, EE reduced oxidative damage markers and selectively increased SOD activity, while other antioxidant systems remained unchanged.

**Conclusion:**

Collectively, all regions exhibited reduced protein oxidative damage in our female-inclusive study design, but through different patterns: a dissociated hippocampal profile (increased TBARS with reduced protein oxidation and higher SOD/sulfhydryls), non-enzymatic thiol-based buffering in the hypothalamus, and SOD-centered defense in the brainstem, supporting a context-dependent model of oxidative regulation.

## 1 Introduction

The central nervous system (CNS) constitutes a complex and functionally specialized network that integrates sensory, cognitive, and motor processes (Asan et al., 2022). Cortical and subcortical regions exhibit distinct functions, with the hippocampus and hypothalamus playing key roles in memory, endocrine regulation, and homeostatic maintenance, while the brainstem coordinates vital autonomic and motor functions, linking the spinal cord to higher brain centers (Zhang et al., 2026).

This functional specialization reflects regional differences in neuronal and glial composition, molecular architecture, and neurotransmitter and synaptic protein profiles. Transcriptomic and multi-omics studies reveal region-specific molecular signatures and distinct gene expression patterns corresponding to anatomical subdivisions and cellular populations, conferring differential vulnerability to physiological and pathological stimuli (Ko et al., 2013; Collado-Torres et al., 2019; Dong et al., 2024).

The brain is uniquely susceptible to oxidative stress, a vulnerability stemming from its high metabolic demand, enrichment in polyunsaturated fatty acids within neuronal membranes, and relatively modest catalase activity, compounded by the unrelenting generation of superoxide as a byproduct of mitochondrial oxidative phosphorylation (Halliwell, 2006; Cobley et al., 2018; Trofin et al., 2025). Superoxide is dismutated into H_2_O_2_ by superoxide dismutase (SOD), which is subsequently scavenged by catalase, glutathione peroxidases, and peroxiredoxins (Sies, 2017).

Glutathione (GSH), the primary low-molecular-weight thiol antioxidant in the central nervous system, plays a pivotal role in safeguarding neuronal membranes against lipid peroxidation and facilitating protein repair via thiol-disulfide exchange. Consequently, the GSH/GSSG ratio remains a hallmark indicator of cellular redox homeostasis (Dringen, 2000; Schulz et al., 2000). Furthermore, protein-bound sulfhydryl (-SH) groups provide a critical layer of defense, acting as redox switches that fine-tune enzyme activity in response to oxidative fluctuations (Aksenov and Markesbery, 2001).

Regional heterogeneity also shapes susceptibility to oxidative stress, driven by high metabolic demand, polyunsaturated lipid content, and redox-active metals, which favor the formation of radicals capable of damaging proteins, lipids, and DNA (Battelli et al., 2014; Shichiri, 2014; Asan et al., 2022). Different brain regions exhibit distinct profiles of reactive oxygen species (ROS), glutathione (GSH), and lipid peroxidation: the hippocampus and amygdala show early and intense oxidative responses; the prefrontal cortex, intermediate responses; and the cerebellum and brainstem, more variable responses depending on the type of oxidative insult (Candelario-Jalil et al., 2001; Vinokurov et al., 2021).

Under physiological conditions, ROS and reactive nitrogen species (RNS) participate in cellular signaling; however, an imbalance relative to antioxidant systems promotes oxidative damage and neuronal dysfunction, contributing to neurodegenerative diseases (Trofin et al., 2025). Major determinants of oxidative stress include mitochondrial dysfunction, such as failures in the electron transport chain, fission/fusion imbalance, and calcium dysregulation; neuroinflammation; and extrinsic factors, including pollutants, trauma, and psychological stress (Picca et al., 2020; Roy and D’Angiulli, 2024; de Araújo et al., 2026).

To counteract these insults, the CNS mobilizes endogenous neuroprotective pathways, including the nuclear factor erythroid 2–related factor 2 (Nrf2)–Antioxidant Response Element (ARE) axis and the phosphoinositide 3-kinase (PI3K)/protein kinase B (Akt) pathway, which regulate antioxidant gene expression and promote cell survival. Proteins such as DJ-1 (Parkinsonism-associated protein 7, PARK7) and paraoxonase 2 (PON2) play key roles in modulating these responses, while hormonal factors further influence their efficacy, collectively contributing to sex-specific differences (Giordano et al., 2011; Ravishankar et al., 2016; Bai et al., 2021).

Environmental enrichment (EE) emerges as a non-pharmacological intervention capable of integrating physical, cognitive, sensory, and social stimuli, inducing structural and functional changes, including synaptic plasticity, neurogenesis, and modulation of antioxidant and inflammatory pathways, thereby enhancing neural adaptability and resilience (Diamond et al., 1964; Kempermann et al., 1997; Van Praag et al., 2000). Typical protocols involve larger housing, social interaction, and diverse objects that stimulate exploration and complex information acquisition, essential for cognitive and behavioral benefits (Rosenzweig et al., 1978; Nithianantharajah and Hannan, 2006; Veissier et al., 2024).

Exposure to multimodal stimuli activates antioxidant pathways, such as Nrf2-ARE, increases superoxide dismutase (SOD) and catalase activity, reduces lipid peroxidation, improves mitochondrial dynamics, modulates inflammatory markers, regulates the hypothalamic-pituitary-adrenal axis, influences microglial activity and cytokine signaling, and enhances brain-derived neurotrophic factor (BDNF) expression, neurogenesis, and cerebral blood flow, thereby promoting neuroprotection (Xu et al., 2016; Ruitenberg et al., 2017; Barros et al., 2019; Zhang et al., 2021; Zhou et al., 2022; Jia et al., 2023; Ramos et al., 2024).

When applied during critical developmental periods, such as adolescence, EE leverages CNS plasticity to enhance adaptive responses and consolidate neural resilience (Andersen, 2003; Borba et al., 2021; Chelini et al., 2022). Early interventions are more effective in reversing maladaptive responses to initial stressors, and even brief protocols can confer long-lasting benefits (Borba et al., 2021). However, the capacity of short-term EE to reshape redox balance and neural plasticity in young females, particularly in the hippocampus, hypothalamus, and brainstem, remains poorly understood, representing a critical gap in our understanding of CNS adaptation. Importantly, most studies investigating EE-mediated oxidative modulation have focused on male animals, pathological models, or prolonged interventions, leaving the effects of short-term EE during female neurodevelopment insufficiently characterized. Moreover, little is known regarding whether anatomically and functionally distinct brain regions engage convergent or divergent antioxidant patterns in response to enrichment. Herein, we hypothesized that short-term EE would induce region-specific redox alterations across the hippocampus, hypothalamus, and brainstem, characterized by differential modulation of oxidative damage markers and antioxidant defenses.

## 2 Materials and methods

### 2.1 Animals and experimental design

All animal procedures were approved by the Ethics Committee on the Use of Animals of the Federal University of Pernambuco under protocol number 0028/2024 and conducted in accordance with international standards for animal research, including the Animals Act 1986, Directive 2010/63/EU, and the Guide for the Care and Use of Laboratory Animals (National Research Council). Female C57BL/6 mice (*n* = 14) obtained from the breeding and experimental vivarium of the Keizo Asami Institute at the Federal University of Pernambuco (ILIKA/UFPE) were used. The animals were maintained under controlled conditions (22 ± 2 °C; 12 h light/dark cycle), with *ad libitum* access to water and standard commercial chow (Nuvilab CR-1, Quimtia, Brazil). At 35 days of age, the animals were randomly assigned to the experimental groups: standard environment (SE; *n* = 7, 3–4 mice per cage; body weight: 16.23 g ± 5.30 g) and enriched environment (EE; *n* = 7; 7 mice per cage; body weight: 16.62 g ± 5.81 g) (Eduarda da Silva Fidélis et al., 2025). In female C57BL/6 mice, the P23–P60 window used here spans the adolescence phase in mice, a phase of high experience-dependent plasticity (Arellano et al., 2024).

### 2.2 Environmental enrichment protocol

**FIGURE 1:**
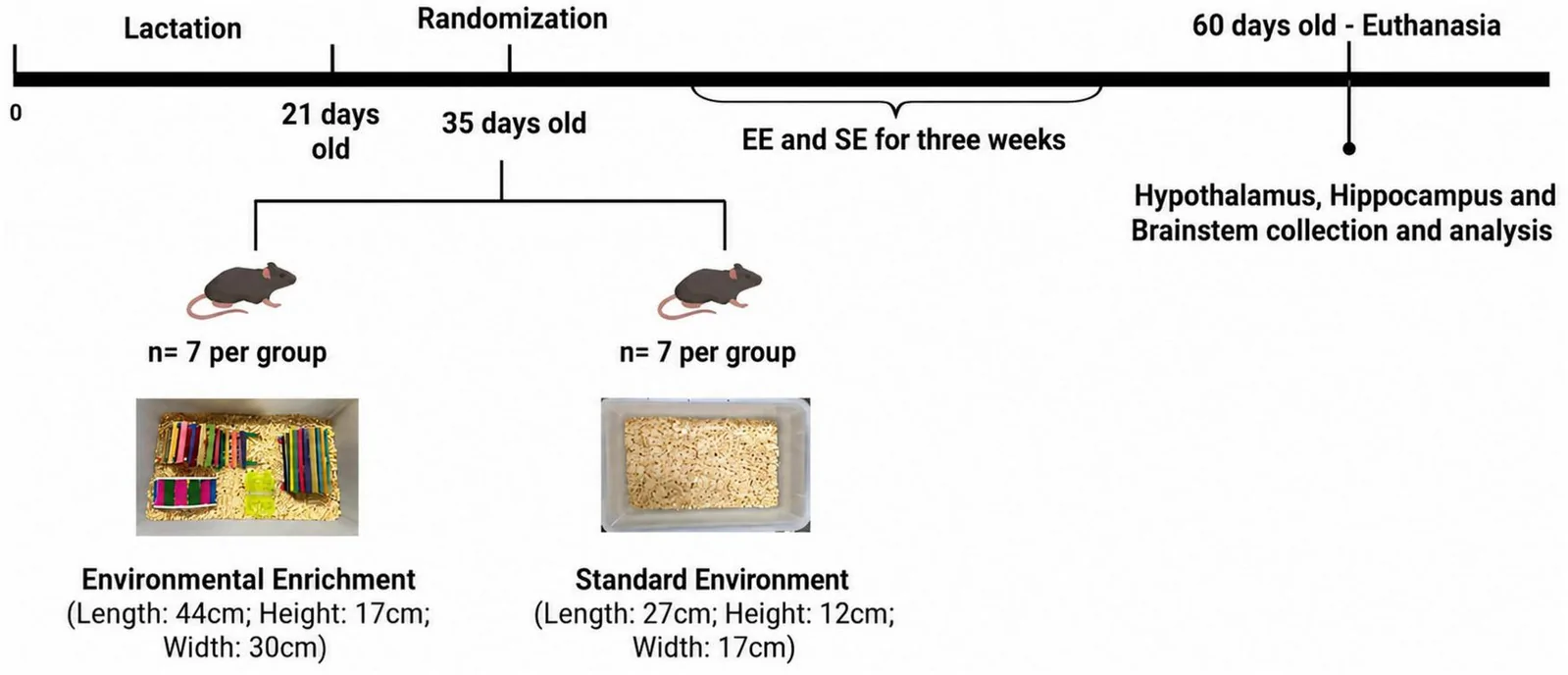
Experimental design of a three-week Environmental Enrichment protocol in female C57BL/6 mice, with collection of hippocampus, hypothalamus, and brainstem for analysis.

The EE protocol was standardized for C57BL/6 mice and consisted of introducing inanimate objects designed to promote sensory, cognitive, and motor stimulation. The environment included tunnels, ladders, plastic and wooden manipulable objects, and running wheels, creating a dynamic and multimodal setting. To ensure environmental variability and complexity, these elements were reorganized weekly, with a progressive increase in both quantity and diversity of arrangements over the three-week intervention period. In line with the literature, animals were housed in larger cages (44 × 30 × 17 cm), allowing higher housing density and increased opportunities for social interaction compared to the SE (27 × 17 × 12 cm). The experimental design is presented in Figure 1.

### 2.3 Euthanasia and brain region dissection

At 60 days of age, the animals were euthanized by intraperitoneal injection of a ketamine hydrochloride (0.100 mg/kg) and xylazine (10 mg/kg) mixture. Immediately after euthanasia, the brain was rapidly removed, and the hippocampus, hypothalamus, and brainstem were carefully dissected based on anatomical landmarks. All samples were immediately collected and stored at –80°C until further analyses.

### 2.4 Tissue homogenate preparation and protein determination

Each dissected brain region (hippocampus, hypothalamus, and brainstem) was individually homogenized in extraction buffer containing 100 mM Tris base (pH 7.5), 10 mM EDTA, 0.1% NP-40, and a protease inhibitor. The homogenates were centrifuged at 461 × *g* for 5 min at 4 °C, and the supernatants were collected. Protein concentrations were determined spectrophotometrically at 595 nm according to the Bradford method (He, 2011). The assay is based on the formation of a protein–Coomassie Brilliant Blue complex. Protein concentrations were determined using a standard curve generated with 1% bovine serum albumin (BSA) as the reference standard. A single homogenate was prepared from each brain region of each animal and subsequently aliquoted to ensure that all biochemical analyzes were performed using the same homogenate preparation (Eduarda da Silva Fidélis et al., 2025; Pedroza et al., 2019).

### 2.5 Oxidative balance

#### 2.5.1 Thiobarbituric acid reactive substances (TBARS)

Lipid peroxidation was assessed by measuring TBARS according to the method described by Buege and Aust (1978). Aliquots of hippocampus, hypothalamus, and brainstem homogenates were mixed with 30% trichloroacetic acid (TCA) and 3 mM Tris–HCl buffer (pH 7.4), followed by centrifugation at 3,000 rpm for 10 min. The supernatant was then incubated with 0.73% thiobarbituric acid (TBA), which reacts with lipid peroxidation products to form a pink chromogen. The reaction was conducted at 100 °C for 15 min. After cooling, absorbance was measured at 535 nm. Results were expressed as nanomoles per milligram (nmol/mg) of protein (Buege and Aust, 1978).

#### 2.5.2 Carbonyls assay

Protein carbonyl levels were determined according to Reznick and Packer (1994). Briefly, 300 μg of protein were precipitated with 30% (w/v) trichloroacetic acid (TCA) and centrifuged at 1,180 × *g* for 14 min. The pellet was resuspended in 10 mM 2,4-dinitrophenylhydrazine (DNPH) and incubated in the dark at room temperature for 1 h, with gentle agitation every 15 min. Samples were washed and centrifuged three times with an ethyl acetate/ethanol solution to remove excess reagent. The final pellet was dissolved in 6 M guanidine hydrochloride and incubated at 37 °C for 30 min. Absorbance was measured at 370 nm, and results were expressed as nanomoles per milligram (nmol/mg) of protein, (Reznick and Packer, 1994).

#### 2.5.3 Superoxide dismutase

SOD activity was determined based on the inhibition of adrenaline autoxidation, according to Misra and Fridovich (1972). The assay was performed in a 1 mL quartz cuvette containing 0.1 M carbonate buffer (pH 10.2), 0.1 mM EDTA, and the sample. The reaction was initiated by the addition of adrenaline (150 mM), and the increase in absorbance due to adrenochrome formation was monitored at 480 nm for 90 s at 30 °C. One unit of SOD activity was defined as the amount of enzyme required to inhibit 50% of the rate of adrenaline autoxidation. Results were expressed in (U/mg) of protein) (Misra and Fridovich, 1972).

#### 2.5.4 Catalase activity

Catalase activity was determined by measuring the rate of hydrogen peroxide (H_2_O_2_) decomposition, monitored by the decrease in absorbance at 240 nm. The reaction mixture consisted of 50 mM phosphate buffer (pH 7.0) and 0.3 mM H_2_O_2_, and the reaction was initiated by adding the biological sample. Changes in absorbance were recorded continuously for 3 min at 20 °C. Enzyme activity was calculated based on the rate of H_2_O_2_ decomposition and expressed as millimoles of H_2_O_2_ consumed. Data were expressed as expressed in (U/mg) of protein (Aebi, 1984).

#### 2.5.5 Glutathione S-transferase (GST)

Glutathione S-transferase activity was determined according to Habig et al. (1974). The assay was performed in a reaction mixture containing 0.1 M potassium phosphate buffer (pH 6.5), 1 mM EDTA, 1 mM reduced glutathione, the sample, and 1 mM 1-chloro-2,4-dinitrobenzene (CDNB). GST activity was measured by monitoring the formation of 2,4-dinitrophenyl-S-glutathione (DNP-SG) at 340 nm at 30°C and expressed as in (U/mg) of protein (Habig et al., 1974).

#### 2.5.6 Non-enzymatic antioxidant response

GSH levels were determined according to the method described by Hissin and Hilf (1976). Samples were diluted (1:10) in 0.1 M phosphate buffer containing 5 mM EDTA (pH 8.0) and incubated with o-phthaldialdehyde (OPT; 1 mg/mL) at room temperature for 15 min. Fluorescence was measured using excitation and emission wavelengths of 350 nm and 420 nm, respectively. Oxidized glutathione (GSSG) levels were determined after derivatization of GSH with 40 mM N-ethylmaleimide for 30 min at room temperature, followed by the addition of 100 mM NaOH. Results were expressed as micromoles per milligram of protein (μmol/mg protein), and cellular redox status was assessed by calculating the GSH/GSSG ratio (Hissin and Hilf, 1976).

#### 2.5.7 Sulfhydryl groups

Total and protein sulfhydryl group levels were determined as described by Aksenov and Markesbery (2001). In this assay, thiol groups react with 5,5’-dithiobis-(2-nitrobenzoic acid) (DTNB), forming a yellow-colored chromophore, 5-thio-2-nitrobenzoic acid. The reaction was performed using homogenates containing 200 μg of protein, and absorbance was measured spectrophotometrically at 412 nm. Results were expressed as millimoles of sulfhydryl groups per milligram of protein (mM/mg protein) (Aksenov and Markesbery, 2001).

### 2.6 Statistical analysis

Sample size was determined based on previous studies evaluating oxidative biomarkers following EE interventions and considering ethical principles of animal reduction. A priori power analysis indicated that *n* = 7/group would detect large effect sizes (Cohen’s d > 1.2) with 80% power at α = 0.05.

**FIGURE 2:**
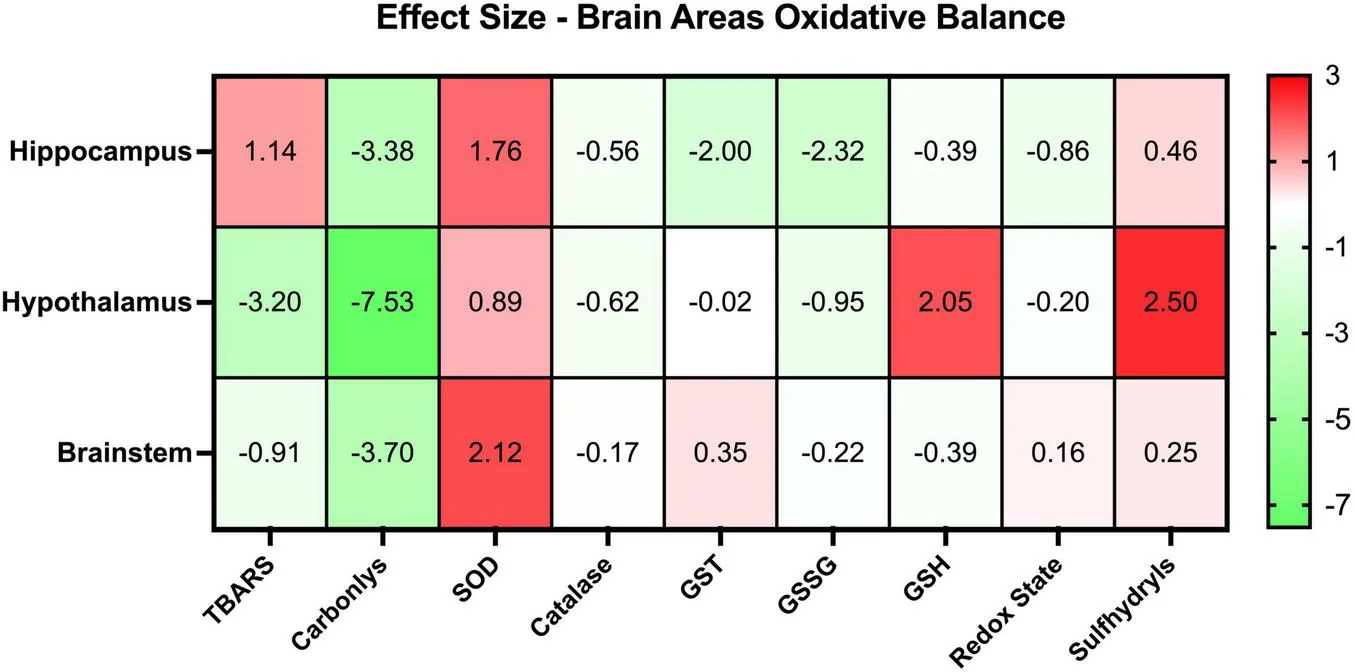
Heatmap with effect size after 3 weeks of Environmental Enrichment in adolescent female mice. Oxidative Balance Markers: Malonaldehyde; Carbonyls; Superoxide Dismutase (SOD); Catalase; Glutathione S Transferase (GST); Oxidized Glutathione (GSSG); Reduced Glutathione (GSH); Redox State (GSH/GSSG ratio); Sulfhydryl’s in the hippocampus, hypothalamus and brainstem, Ignored (0.0 to < 0.2), small ( ≥ 0.2 to < 0.5), moderate ( ≥ 0.5 to < 0.8), large ( ≥ 0.8 to < 1.3), and very large ( ≥ 1.3).

Data normality was evaluated using the Shapiro–Wilk test. Results were presented as mean ± standard deviation (SD). Comparisons between two experimental groups were conducted using the unpaired Student’s *t*-test. Effect size was calculated using Hedges’ g, based on group means, standard deviations, and sample size. The magnitude of the effect size was interpreted as negligible (0.0 to < 0.2), small ( ≥ 0.2 to < 0.5), moderate ( ≥ 0.5 to < 0.8), large ( ≥ 0.8 to < 1.3), or very large ( ≥ 1.3), as shown in Figure 2. Statistical significance was set at *p* < 0.05. All analyses were performed using GraphPad Prism version 10 (GraphPad Software Inc., La Jolla, CA, USA).

## 3 Results

Effects of environmental enrichment on the oxidative balance of brain areas.

### 3.1 Hippocampus

**FIGURE 3:**
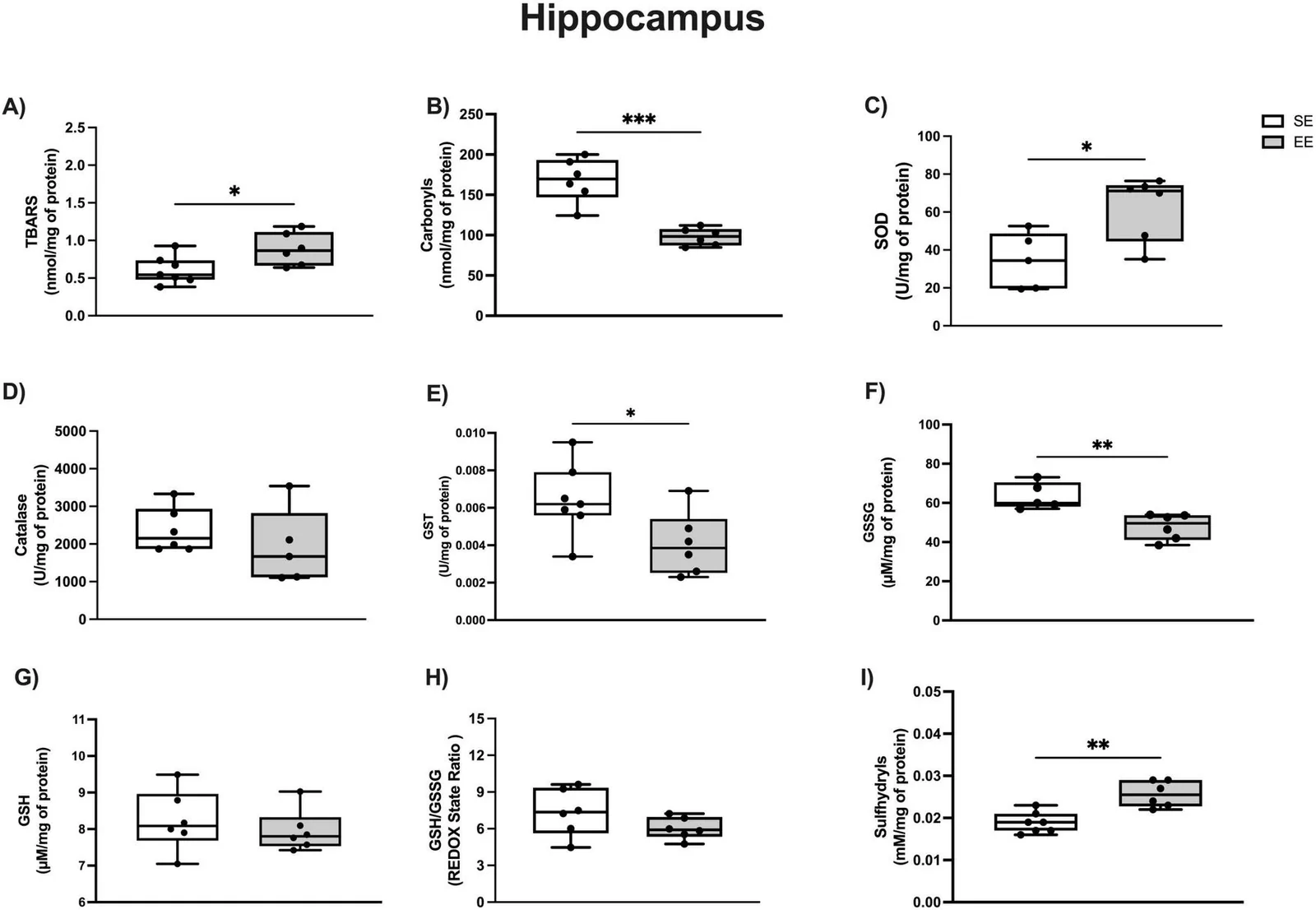
Effects of Environmental Enrichment on the oxidative balance of the hippocampus. Data are presented as box-and-whisker plots. The center line indicates the median, the box represents the interquartile range, and the whiskers indicate the minimum and maximum values (*n* = 5–**7** mice/analysis). **(A)** TBARS, **(B)** Carbonyls, **(C)** SOD, **(D)** Catalase, **(E)** GST, **(F)** GSSG, **(G)** GSH, **(H)** GSH/GSSG, and **(I)** Sulfhydryls. Differences were assessed using the unpaired Student’s *t*-test. *p < 0.05; **p < 0.01; ***p < 0.001. EE, Environmental Enrichment; SE, Standard Environment.

We first investigated the impact of a three-week EE protocol on hippocampal oxidative stress markers. Regarding lipid peroxidation, animals exposed to EE exhibited a significant increase in TBARS levels compared to the SE group (*p* = 0.03; Figure 3A). Conversely, protein oxidation was significantly attenuated by the intervention, as evidenced by a marked reduction in protein carbonyl levels in the EE group (*p* = 0.0008; Figure 3B).

Next, we assessed the enzymatic antioxidant defenses, including SOD, catalase, and GST activities. Our findings revealed that 3 weeks of EE significantly upregulated hippocampal SOD activity compared to the SE group (*p* = 0.01; Figure 3C). In contrast, no significant alterations were detected in catalase activity (*p* = 0.40; Figure 3D). Interestingly, GST activity was significantly attenuated in EE-exposed animals compared to the SE group (*p* = 0.03; Figure 3E).

Non-enzymatic antioxidant defenses, including GSSG, GSH, and total sulfhydryls, were also modulated by the three-week EE protocol. Specifically, GSSG levels were significantly reduced in the EE group relative to SE controls (*p* = 0.004; Figure 3F). In contrast, no significant changes were detected in GSH levels (*p* = 0.51; Figure 3G) or the GSH/GSSG ratio (*p* = 0.17; Figure 3H). Notably, EE-exposed animals exhibited a significant elevation in total sulfhydryl content (*p* = 0.001; Figure 3I), further indicating an enhanced non-enzymatic buffering capacity in the hippocampus.

### 3.2 Hypothalamus

**FIGURE 4:**
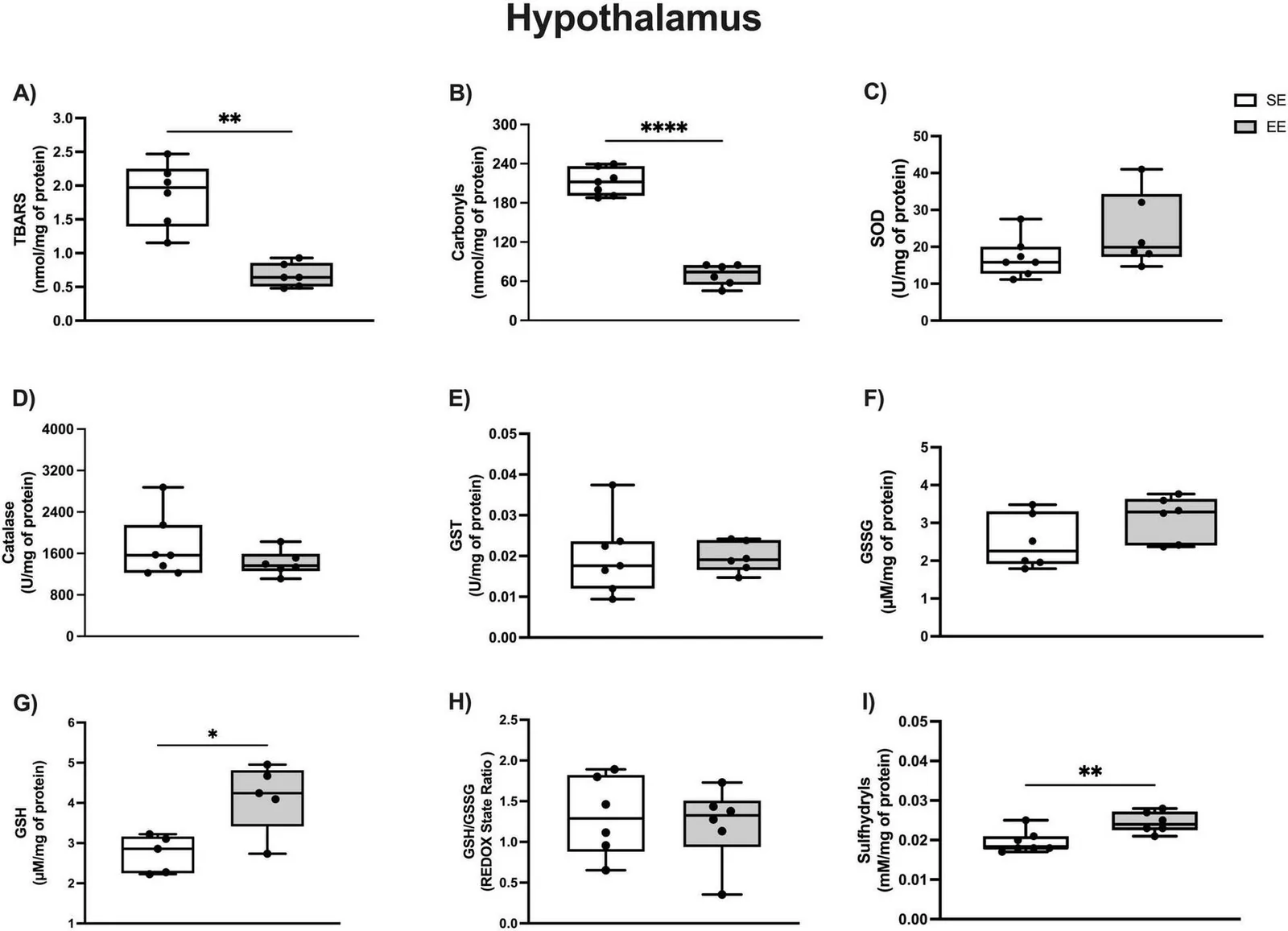
Effects of Environmental Enrichment on the oxidative balance of the hypothalamus. Data are presented as box-and-whisker plots. The center line indicates the median, the box represents the interquartile range, and the whiskers indicate the minimum and maximum values (*n* = 6–7 mice/analysis). **(A)** TBARS, **(B)** Carbonyls, **(C)** SOD, **(D)** Catalase, **(E)** GST, **(F)** GSSG, **(G)** GSH, **(H)** GSH/GSSG, and **(I)** Sulfhydryls. Differences were assessed using the unpaired Student’s *t*-test. *p < 0.05; **p < 0.01; ****p < 0.0001. EE, Environmental Enrichment; SE, Standard Environment.

In the hypothalamus, the three-week EE protocol exerted a robust protective effect against oxidative damage. Lipid peroxidation, assessed via TBARS levels, was significantly reduced in the EE group compared to SE animals (*p* = 0.001; Figure 4A). Similarly, protein carbonyl levels were markedly lower following EE exposure, indicating a significant reduction in protein oxidation (*p* < 0.0001; Figure 4B). Regarding enzymatic antioxidant defenses, no significant alterations were observed in the activities of SOD (*p* = 0.16; Figure 4C), catalase (*p* = 0.26; Figure 4D), or GST (*p* = 0.96; Figure 4E) after the intervention.

In contrast, the non-enzymatic antioxidant profile was significantly modulated in this region. While GSSG levels remained unchanged (*p* = 0.13; Figure 4F), GSH levels were significantly elevated in the EE group (*p* = 0.01; Figure 4G). Although the GSH/GSSG ratio showed no statistical difference between groups (*p* = 0.73; Figure 4H), EE-exposed animals exhibited a significant increase in total sulfhydryl content (*p* = 0.007; Figure 4I), further reinforcing evidence of enhanced hypothalamic antioxidant capacity.

### 3.3 Brainstem

**FIGURE 5:**
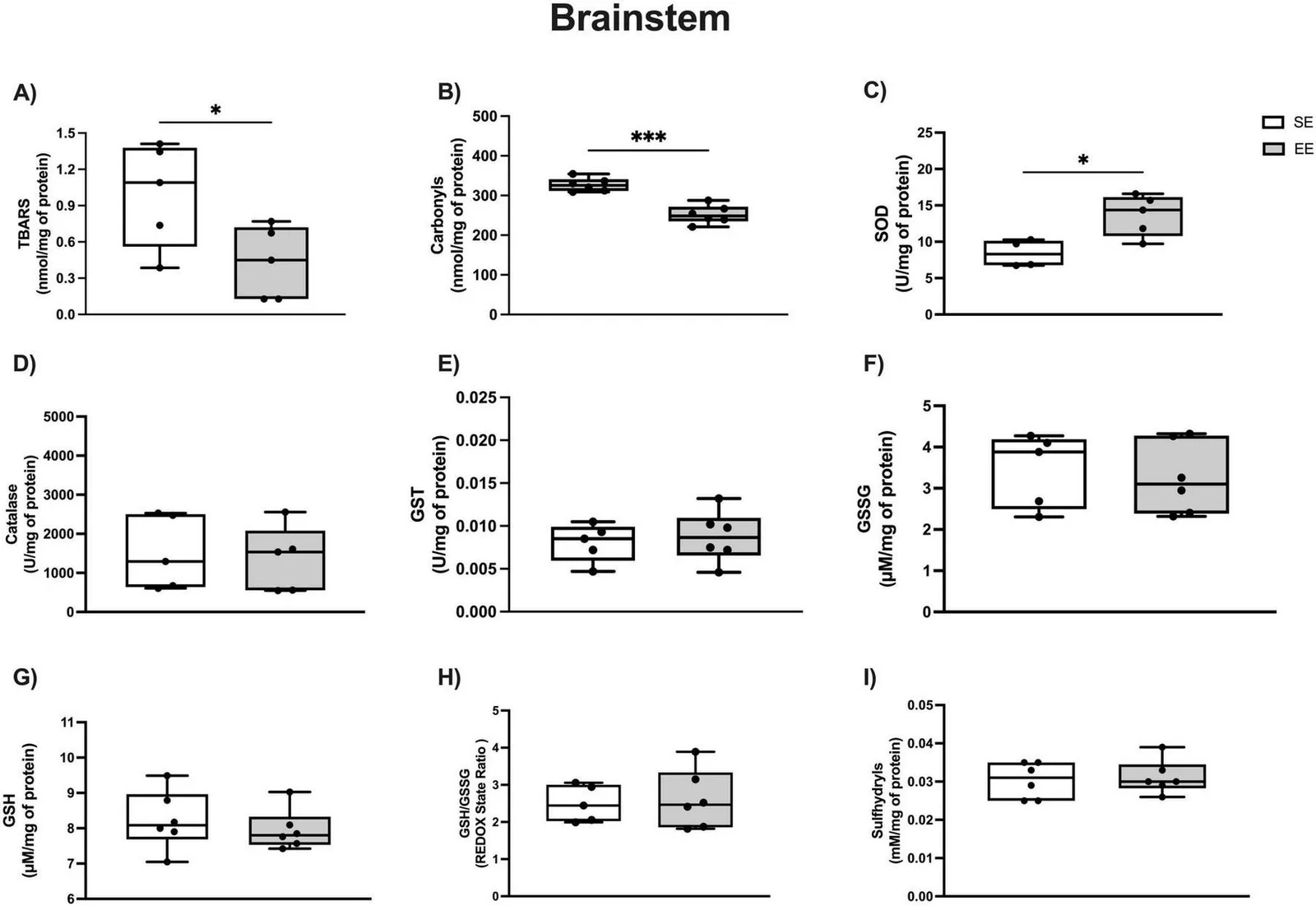
Effects of Environmental Enrichment on the oxidative balance of the brainstem. Data are presented as box-and-whisker plots. The center line indicates the median, the box represents the interquartile range, and the whiskers indicate the minimum and maximum values (*n* = 4–6 mice/ analysis). **(A)** TBARS, **(B)** Carbonyls, **(C)** SOD, **(D)** Catalase, **(E)** GST, **(F)** GSSG, **(G)** GSH, **(H)** GSH/GSSG, and **(I)** Sulfhydryls. Differences were assessed using the unpaired Student’s *t-*test. *p < 0.05; ***p < 0.001. EE, Environmental Enrichment; SE, Standard Environment.

Consistent with findings from other regions, the three-week EE protocol significantly mitigated oxidative damage in the brainstem. Lipid peroxidation, as measured by TBARS levels, was significantly reduced in the EE group compared to SE animals (*p* = 0.04; Figure 5A). Furthermore, EE exposure led to a marked decrease in protein carbonyl levels, indicating a robust reduction in protein oxidation (*p* = 0.0001; Figure 5B). Regarding enzymatic defenses, a significant upregulation in SOD activity was observed in the EE-exposed animals (*p* = 0.01; Figure 5C).

In contrast, other enzymatic and non-enzymatic antioxidant markers remained largely unaffected by the intervention in this region. No significant differences were detected in the activities of catalase (*p* = 0.79; Figure 5D) or GST (*p* = 0.66; Figure 5E). Similarly, the non-enzymatic profile showed no statistical alterations, with GSSG (*p* = 0.72; Figure 5F) and GSH levels (*p* = 0.51; Figure 5G) remaining stable between groups. Consequently, the GSH/GSSG ratio, reflecting the local redox status, showed no significant variation (*p* = 0.78; Figure 5H). Finally, total sulfhydryl content was not significantly altered following the three-week EE protocol (*p* = 0.75; Figure 5I).

Our results are summarized in Table 1. The magnitude of the effect size, which was interpreted as negligible (0.0 to < 0.2), small ( ≥ 0.2 to < 0.5), moderate ( ≥ 0.5 to < 0.8), large ( ≥ 0.8 to < 1.3), or very large ( ≥ 1.3), is shown in Figure 2.

**TABLE 1:** Summary of the effects of short-term environmental enrichment on brain redox status in adolescent female mice.

Variable	Hippocampus	Hypothalamus	Brainstem
TBARS (nmol/mg protein)	↑	↓	↓
Carbonyls (nmol/mg protein)	↓	↓	↓
SOD (U/mg protein)	↑	↔	↑
Catalase (U/mg protein)	↔	↔	↔
GST (U/mg protein)	↓	↔	↔
GSSG (μmol/mg protein)	↓	↔	↔
GSH (μmol/mg protein)	↔	↑	↔
GSH/GSSG (μmol/mg protein)	↔	↔	↔
Sulfhydryls (mM/mg protein)	↑	↑	↔

GST, Glutathione S Transferase; GSSG, Oxidized Glutathione; GSH, Reduced Glutathione; SOD, Superoxide Dismutase. ↑ Significant increase; ↓ Significant decrease; ↔ No significant difference between the SE and EE groups.

## 4 Discussion

Environmental enrichment (EE) is widely established as a potent non-pharmacological intervention that promotes neuroplasticity, resilience, and behavioral modulation (Nithianantharajah and Hannan, 2006; Kempermann et al., 1997). The present study provides evidence that a three-week EE protocol can modulate specific redox markers in a region-dependent manner across three functionally distinct brain regions (hippocampus, hypothalamus, and brainstem) in adolescence female C57BL/6 mice. Although EE consistently attenuated protein carbonyl content across all examined areas, the divergent patterns of lipid peroxidation, enzymatic activities, and non-enzymatic thiol-based defenses revealed a complex, region-specific redox signature. These findings *(i)* underscore the pivotal role of anatomical and neurochemical contexts in orchestrating redox modulation to environmental stimuli and *(ii)* support a context-dependent model of oxidative stress that aligns with the emerging framework of oxidative eustress.

The hippocampal response following 3 weeks of EE is the most intricate among the analyzed regions by displaying a distinct dual redox signature. While animals exposed to enrichment exhibited elevated TBARS levels as a classical biomarker of lipid peroxidation associated with malondialdehyde and other reactive aldehydes, there was a simultaneous and significant reduction in protein carbonylation. This profile was accompanied by a partial and selective modulation of the antioxidant defenses assessed, characterized by increased SOD activity, an expansion of the protein sulfhydryl pool, and a decline in GSSG content, whereas catalase activity was unchanged. Because glutathione peroxidase and glutathione reductase were not measured, these observations describe a limited, selective adjustment rather than a global reinforcement of antioxidant capacity.

One possible interpretation is that the increased TBARS reflects controlled, signaling-level ROS production (oxidative eustress) rather than oxidative injury. However, because TBARS is a non-specific index of lipid peroxidation and signaling ROS were not directly measured, this interpretation remains speculative. The isolated increase in hippocampal TBARS may instead reflect heightened synaptic and metabolic activity together with activity-dependent membrane lipid remodeling during EE, rather than net oxidative damage. Thus, the source and biological significance of the increased TBARS cannot be established from the present data and should be interpreted cautiously. Future studies incorporating direct ROS measurements together with more specific lipid peroxidation markers, such as 4-hydroxynonenal (4-HNE), and assessment of potential upstream sources, including NADPH oxidases, will be necessary to determine whether these findings truly reflect adaptive redox signaling.

In this framework, moderate ROS concentrations serve as critical intracellular signaling mediators, supporting processes such as neuroplasticity, synaptic transmission, and the maintenance of neuronal homeostasis (Radak et al., 2008; Sies et al., 2017; Nitti et al., 2022; Caporossi et al., 2026).

The increase in SOD activity may contribute to this adaptive response by accelerating the dismutation of superoxide radicals into hydrogen peroxide (H_2_O_2_). At physiological concentrations, H_2_O_2_ acts as a molecular trigger that oxidizes specific cysteine sulfhydryl (—SH) residues on Kelch-like ECH-associated protein 1 (Keap1), inducing a conformational change that liberates Nrf2 (Fourquet et al., 2010).

Once spared from cytoplasmic degradation, Nrf2 translocates to the nucleus and binds to the Antioxidant Response Element within the DNA. The activation of the Nrf2–ARE axis orchestrates the induction of enzymes involved in glutathione synthesis and regeneration, the transport of redox precursors, and the neutralization of reactive species to fortify the intracellular antioxidant network. This mechanism provides a plausible biochemical basis for the preservation of protein integrity, evidenced by reduced carbonyls, the rise in sulfhydryl levels, and the shift of the hippocampal redox environment toward a more reductive and resilient state (Harvey et al., 2009; Tonelli et al., 2018).

Taken together, these findings indicate that the hippocampus, under the influence of EE, does not undergo unregulated oxidative damage but rather engages in an adaptive redox remodeling characterized by enhanced antioxidant capacity and efficiency in regulating reactive species. This fine-tuning of the redox environment may represent a central mechanism through which EE promotes greater neural resilience and optimizes both synaptic and cognitive function.

In the hypothalamus, EE induced a redox signature distinct from that observed in the hippocampus, characterized by a reduction in oxidative damage markers, as evidenced by decreased TBARS and protein carbonyl levels. Notably, these protective effects occurred in the absence of significant changes in enzymatic antioxidant activities (SOD, catalase, and GST) or GSSG levels, whereas non-enzymatic defenses were selectively enhanced, as indicated by increased GSH content and total sulfhydryl levels.

The lack of enzymatic upregulation suggests that basal antioxidant capacity was sufficient to sustain redox homeostasis and that the reduction in oxidative damage was primarily mediated by expansion of the non-enzymatic antioxidant pool. This pattern indicates a metabolic redox alteration in which cellular protection is achieved without additional induction of enzymatic systems (Ramos et al., 2024).

From a neuroendocrine perspective, these findings are particularly relevant given the hypothalamus’s central role in regulating the hypothalamic–pituitary–adrenal (HPA) axis, circadian rhythms, and energy balance. This region exhibits a high density of glucocorticoid receptors, rendering it especially sensitive to corticosterone-mediated redox alterations (Spiers et al., 2014). Elevated glucocorticoid levels have been associated with mitochondrial dysfunction, increased glutamate efflux, and enhanced production of ROS, thereby promoting intracellular glutathione consumption and redox imbalance (Popoli et al., 2012; Choi and Han, 2021; Gulyaeva, 2021). Moreover, chronic glucocorticoid exposure may impair the availability of precursors required for GSH synthesis, such as cysteine, ultimately limiting the replenishment of antioxidant reserves (Lewerenz et al., 2013).

Within this framework, the increase in GSH levels observed in EE-exposed animals, together with reduced oxidative damage markers, suggests a state of lower cellular redox demand. The maintenance of GSSG levels alongside increased GSH indicates that the intracellular environment was not under elevated pro-oxidant pressure, allowing preservation and possibly accumulation of this key antioxidant. Although corticosterone levels were not directly measured, this redox profile is consistent with a potential attenuation of basal HPA axis tone, resulting in reduced glucocorticoid-driven oxidative burden. This interpretation aligns with evidence demonstrating that EE reduces HPA axis reactivity and lowers basal corticosterone levels (Smail et al., 2020).

Furthermore, the increase in GSH in the absence of changes in GSSG or the GSH/GSSG ratio suggests that EE may have promoted *de novo* glutathione synthesis or reduced its rate of consumption, rather than enhancing its recycling from GSSG (Lu, 2013; Santacroce et al., 2023). Collectively, these findings reveal a region-specific adaptive pattern. While the hippocampus appears to rely on controlled oxidative signaling to support plasticity, the hypothalamus establishes a more reduced and protected redox environment, which is essential for maintaining neuroendocrine precision and systemic resilience to stress.

In the brainstem, EE elicited a neuroprotective yet conservative response. A significant reduction in TBARS and protein carbonyl levels was observed, concomitant with an increase in SOD activity, without detectable changes in catalase or GST activities, glutathione system components, or sulfhydryl levels. This pattern indicates the recruitment of a primary antioxidant defense centered on SOD, sufficient to mitigate macromolecular oxidative damage without requiring extensive remodeling of the intracellular redox network (Ighodaro and Akinloye, 2018).

The selective upregulation of SOD in this region likely reflects increased metabolic activity within brainstem nuclei, driven by the sensorimotor and autonomic stimulation inherent to EE. Such functional activation promotes superoxide generation, which at physiological levels triggers compensatory SOD induction via redox-sensitive signaling pathways (Powers et al., 2024; Ramos et al., 2024). The resulting reduction in superoxide availability directly limits lipid peroxidation and protein oxidation, processes further amplified by highly reactive downstream species such as the hydroxyl radical (OH∙) and peroxynitrite (ONOO^–^) (Beckman, 1994; Beckman and Koppenol, 1996; Pacher et al., 2007).

The absence of changes in enzymes responsible for H_2_O_2_ detoxification, as well as in glutathione system components, suggests that the H_2_O_2_ generated from increased SOD activity was efficiently buffered by constitutive antioxidant systems, including peroxiredoxins and glutathione peroxidases. These enzymes exhibit high kinetic efficiency in H_2_O_2_ reduction, with second-order rate constants (∼10^7^ M^−1^s^−1^) exceeding those of catalase, thereby obviating the need for additional antioxidant induction (Sies, 2017). Alternatively, H_2_O_2_ may have primarily functioned as a signaling molecule at the concentrations generated, consistent with the concept of oxidative eustress, in which reactive species exert regulatory roles (Sies and Jones, 2020).

The maintenance of GSH, GSSG, and the GSH/GSSG ratio further supports the notion that intracellular redox homeostasis remained preserved, with no evidence of oxidative overload or excessive antioxidant consumption (Forman et al., 2010; Circu and Aw, 2012). This profile indicates that the response induced by EE in the brainstem was sufficient to contain reactive species formation at its early stages, preventing the propagation of oxidative cascades and eliminating the need for additional compensatory mechanisms (Ighodaro and Akinloye, 2018).

This more restricted and efficient pattern may reflect the intrinsic functional nature of the brainstem, a region critical for regulating vital functions, including respiration, cardiovascular activity, and autonomic control, in which redox stability is essential for maintaining systemic homeostasis. In this context, in contrast to the hippocampus, which appears to exploit redox signaling to support plasticity, the brainstem adopts a more conservative pattern, prioritizing tight control of reactive species and preservation of cellular integrity.

The divergent redox signatures identified across the three regions are not contradictory; rather, they collectively reveal regional heterogeneity as a defining hallmark of EE-induced redox remodeling. All three regions showed protection against protein oxidative damage (reduced carbonyls) but achieved this through distinct profiles: the hippocampus via SOD upregulation, thiol preservation, and eustress-associated TBARS signaling; the hypothalamus via non-enzymatic antioxidant expansion (GSH, sulfhydryls); and the brainstem via a focused SOD induction.

Our findings in healthy adolescent animals complement an extensive literature demonstrating that EE exerts protective effects against oxidative damage in neurological disease models. EE has been shown to reduce MDA and carbonyl levels in the hippocampus following stroke (Zhang et al., 2021), reduce 4-HNE in the prefrontal cortex following hypoxia (Zhang et al., 2016), attenuate nitrotyrosine accumulation in an Alzheimer-like model (Herring et al., 2010), and restore antioxidant enzyme activities following traumatic brain injury (Tapias et al., 2022), as reviewed by Ramos et al. (2024). Mechanistically, EE exerts antioxidant effects in pathological contexts through NADPH oxidase 2 (NOX2) inhibition - reducing superoxide at its source - and through Nrf2-ARE pathway activation, upregulating cytoprotective genes including HO-1, NQO1, SOD, and GSH-synthesizing enzymes (Zhang et al., 2016, 2021). In our study of healthy animals, these same pathways appear engaged in a more nuanced, region-specific manner, prioritizing preventive redox optimization over reactive antioxidant induction.

The uniform reduction in protein carbonyls across all three regions - despite divergent lipid-peroxidation and antioxidant-enzyme patterns - suggests that, under this protocol, protection of the protein pool is a shared feature of the enrichment response. In contrast, the route to that protection is region-dependent. This matters because it indicates that region-resolved sampling is necessary: whole-brain or single-region measurements would misrepresent the redox response to enrichment.

Several limitations merit acknowledgment. Firstly, a central limitation of this study is its housing design: all enriched animals were maintained in a single enriched cage, whereas the manipulation was applied at the cage level. The cage is therefore the true experimental unit and analyzing co-housed animals as independent replicates constitutes pseudo replication (Lazic, 2010). The present findings should be read as exploratory and hypothesis-generating, and the between-region patterns require confirmation in designs incorporating multiple independent enrichment cages. Secondly, the study assessed a broad biochemical panel but did not include molecular analyses of upstream regulatory pathways such as Nrf2 nuclear translocation, NADPH oxidase isoform expression, or mitochondrial ROS production rates. Thirdly, the observed changes in GSH levels and SOD activity are downstream functional readouts of redox regulation. However, because such changes can arise from transcriptional, post-transcriptional, and post-translational mechanisms, they cannot be interpreted as direct evidence of altered gene expression; resolving the underlying level of regulation would require targeted molecular analyses. Fourthly, we did not include behavioral, BDNF, corticosterone, or neurogenesis endpoints, so the effectiveness of the enrichment in engaging its canonical central pathways was not independently verified. The redox findings are therefore not anchored to a functional outcome. Fifthly, because the enrichment included running wheels, voluntary physical activity was an inherent component of the paradigm and cannot be disentangled from its cognitive, sensory, and social elements. The redox effects reported here may therefore derive wholly or partly from exercise-driven mechanisms, which are themselves known to modify brain redox status (Csikos et al., 2026; Grégoire et al., 2018).

Additionally, the relatively small sample size and the large number of biochemical comparisons increase the possibility of type I statistical error. Therefore, the present findings should be interpreted as evidence of robust regional trends requiring future validation in larger cohorts and with complementary molecular analyses.

Finally, the exclusive use of female animals limits direct generalizability to male cohorts, and future studies should directly compare sex-specific redox alterations under identical EE conditions. However, the use of exclusively female animals deserves a contextual consideration. Most published EE studies examining cerebral oxidative balance have used male rodents; only one of sixteen studies in the recent systematic review by Ramos et al. (2024) employed female animals. Evidence suggests that female brains may rely on a distinct antioxidant profile as observed under this protocol. For example, female-enriched rats showed protection against protein oxidation and superoxide accumulation comparable to males, but without equivalent activation of all enzymatic antioxidant systems, pointing to an intrinsic non-enzymatic buffering capacity (Marmol et al., 2015).

## 5 Conclusion

Three weeks of EE produced a pattern of region-specific modulation of oxidative balance in the hippocampus, hypothalamus, and brainstem of adolescent female C57BL/6 mice. All three regions showed protection against protein oxidative damage but achieved this through distinct mechanisms: a hippocampal redox pattern characterized by increased TBARS despite reduced protein oxidation and higher SOD activity and sulfhydryl levels; a GSH-dependent, non-enzymatic antioxidant expansion in the hypothalamus; and a focused SOD induction in the brainstem. These findings support the concept that short-term environmental enrichment promotes region-specific redox modulation during the adolescence stage and may contribute to mechanisms associated with neural resilience.

## Data Availability

The raw data supporting the conclusions of this article will be made available by the authors, without undue reservation.
